# Prevalence of Rhesus D negativity among reproductive age women in Southern Ethiopia: a cross-sectional study

**DOI:** 10.1186/s12905-021-01315-3

**Published:** 2021-04-19

**Authors:** Tesfaye K. Kanko, Melat K. Woldemariam

**Affiliations:** 1 Department of Biomedical Sciences, School of Medicine, College of Medicine and Health Sciences, Arba Minch University, Arba Minch, Ethiopia; 2 Department of Medical Laboratory Science, College of Medicine and Health Sciences, Arba Minch University, Arba Minch, Ethiopia

**Keywords:** Blood phenotype, Rhesus D factor, Rh negative, Reproductive women

## Abstract

**Background:**

The Rhesus (Rh) blood group system is the next most clinically significant blood group system following the ABO blood group. Rh D-negative women are at risk of alloimmunization following exposure to Rh D-positive blood. The exposure of Rh D-negative women to Rh D-positive fetal blood may cause hemolytic disease of the fetus or new-born due to Rh incompatibility. Knowing Rh blood phenotype has paramount importance to prevent the risk of sensitization and bad obstetric outcome in Rh D-negative women. Despite the aforementioned fact, the distribution of Rh D-negative phenotype of women was not explored in Arba Minch Zuria district, southern Ethiopia. This study was aimed to assess the prevalence of Rh D-negative blood phenotype among reproductive-age women in Arba Minch Zuria district, southern Ethiopia.

**Methods and materials:**

A community-based cross-sectional study was conducted among reproductive-age women in Arba Minch Zuria district, Southern Ethiopia from March to April 2019. Socio-demographic data were collected using an interviewer-administered semi-structured questionnaire and blood phenotype determination was done by laboratory technicians using the slide method principle aseptically and Statistical Package for Social Science (SPSS) version 21 was used for analysis.

**Result:**

The data were collected from 417 study participants with a 98.8% response rate. This study revealed that 2.1%, 1.9%, 1.2%, and 1% of study participants with blood group O, A, B, and AB were Rh D negative, respectively. In this study, the overall prevalence of Rh D negative phenotype was found 6.2% among reproductive-age women in Arba Minch Zuria district, Southern Ethiopia.

**Conclusions:**

This study showed a high prevalence of Rh D negative factor among reproductive-age women in Arba Minch Zuria district. Therefore, counseling of reproductive age women on the importance of Rh D factor status determination would be worthy to avoid the potential risk of sensitization among Rh D negative women in order to prevent hemolytic disease of the fetus and new-born.

## Introduction

The Rhesus (Rh) blood group system is the next most clinically significant blood group system following the ABO blood group [1, 2]. Rh D antigen is recognized as one of the major blood group antigens such as A and B antigens present on the surface of red blood cells [3, 4].

The distribution of Rh D antigen significantly varies with race. The prevalence of D antigen is higher in Africans and appears to be lower in Asians [5].

Determination of blood group in Rh D-negative pregnant women allows reasonable precautions that limit the risk to the fetus. One of the facts behind fetal loss and death in Rh D-negative mothers is alloimmunization, which causes hemolytic disease of the fetus and new-born [6–8]. Alloimmunization is due to antibody production against specific exogenous D antigens which are introduced in the body during pregnancy [8, 9]. This is particularly of interest in Rh D-negative women of childbearing age due to the possibility of exposure to paternally acquired Rh D positive fetal red cells during pregnancy [6–9].

Transplacental or feto-maternal hemorrhage (FMH) occurs during abortion, ectopic pregnancy, miscarriage, or at delivery and leads to sensitization to the D antigen if the mother is Rh D negative and the fetus is Rh D positive [9, 10]. Once sensitization has occurred, it is irreversible [10]. This sensitization may produce immunological memory in the mother for future pregnancies [11]. Rhesus incompatibility during pregnancy is one of the major determinants for perinatal morbidity and mortality [12, 13]. Therefore, this study was aimed to assess the prevalence of Rh D-negative blood phenotype among reproductive-age women in Arba Minch Zuria district, southern Ethiopia. The findings of this study would be important for the prevention and control of hemolytic disease of the fetus and new-born among Rh D negative women during pregnancy.

## Methods and materials

### Study setting, design and population

A community-based cross-sectional study was conducted among reproductive-age women (women within the age range of 15–49 years) in Arba Minch Zuria district from March to April 2019. Arba Minch Zuria district is one of the districts in the Gamo Zone of the southern part of Ethiopia. According to the Central Statistics Agency (CSA) of Ethiopia, this district has a total population of 164,529, of whom 82,199 are men and 82,330 women; none of its population are urban dwellers [14].

### Sample size determination

The sample size was calculated using a single population proportion formula with the estimated prevalence of knowledge of Rh disease as 50% among reproductive age women in Arba Minch Zuria district, with 5% marginal error(d) and a confidence interval of 95% (Zα/2 = 1.96). Based on these assumptions and adding a 10% nonresponse rate, the total estimated sample size was 422.

### Inclusion and exclusion criteria

Women in the reproductive age group (15–49 years old) and who lived for at least six months in the selected kebeles were included. Women who have difficulty of communication (hearing problems) and who were severely ill during data collection were excluded.

### Sampling technique

A multistage sampling method was utilized to select study participants from Arba Minch Zuria district. Arba Minch Zuria district has 29 kebele administrations. From the total kebeles in the district, six kebeles were selected randomly. Then a census was done to identify the number of reproductive-age women in the six selected kebeles. After this, the proportionate allocation method was applied to determine the required number of study participants from each selected kebele using the number of reproductive-age women obtained from the census done at each kebele administration. Finally, the study participants were selected randomly and data collection was conducted from each selected kebele.

### Data collection methods

Data regarding socio-demographic variables such as age, marital status, educational status, occupation, and religion of the participants were collected using an interviewer-administered semi-structured questionnaire. Six trained laboratory technicians were used for data collection and the data collection task was supervised closely by the investigators. The determination of the ABO blood group and Rh blood group test was done according to the principle of slide method by a laboratory technician. A drop of blood (approx. 40 µL) from each study participant was placed on a glass slide in three places. For monoclonal blood grouping antibodies, in vitro diagnostic reagents of Tulip Diagnostics Private Limited were used. A drop of each of the antisera, A, B, and D was added and mixed with each blood drop, with the aid of an applicator stick. Then, the mixture was rocked gently for 2 min to observe for agglutination. The results of agglutination were recorded immediately after waiting for two minutes. The agglutination in blood drop A was considered as group A and agglutination in blood drop B as group B. The agglutination in both drops was considered as group AB, and if both blood drops were not agglutinating, it was considered as group O. The agglutination in blood drop D was considered as Rh-D positive whereas non-agglutination in blood drop D was considered as Rh-D negative.

### Data quality control

Two days of training were given for data collectors about the objectives of the study, interview technique, context of the questionnaire, procedure, and interpretation of the slide method for blood group determination tests. Data collection was closely supervised by investigators. For data quality control, the collected data were sorted as filled or incompletely filled. The quality of the reagent or procedure was maintained by running a known positive and negative control sample.

### Data processing and analysis

Data were coded and entered into Epi info version 3.5.1 and exported to SPSS version 21 for statistical analysis. Descriptive statistics were used to present the data. Descriptive statistics like frequency and percentage were used to summarize the socio-demographic characteristics of the study participants and their blood group. Tables, figures, and narrations were used to present the findings.

## Results

### Sociodemographic characteristics of the study participants

The data were collected from 417 study participants with a 98.8% response rate. Regarding age distribution, 199 (47.7%) of the respondents were within the age range of 15–24 years. In terms of marital status, the majority of the participants were married and concerning educational status, more than half of the participants attended primary school. Looking at their employment status from the total participants, 219 (52.5%) were housewives. Concerning the participant’s religion, about 340 (81.5%) respondents were protestant religion followers as shown in (Table 1).

**Table 1:** Sociodemographic characteristics of the study participants in Arba Minch Zuria district (n = 417)

Variables	Frequency (n)	Percentage (%)
Age		
15–24	199	47.7
25–34	169	40.5
35–44	47	11.3
45–49	2	0.5
Marital status		
Single	121	29
Married	294	70.5
Divorced	2	0.5
Educational status		
Illiterate	65	15.6
Read and write	16	3.8
Primary school	223	53.5
Secondary school	83	19.9
College and above	30	7.2
Occupation		
Farmer	22	5.3
Gov’t employee	16	3.8
Housewife	219	52.5
Merchant	50	12
Student	110	26.4
Religion		
Orthodox Tewahido	77	18.5
Protestant	340	81.5

### Blood group profile of the study population

**Fig. 1:**
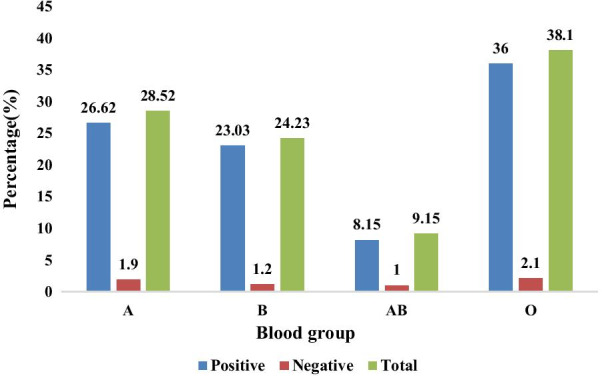
ABO blood group profile among study participants in Arba Minch Zuria district (n = 417)

Regarding the blood group profile, among the total study population, 159 (38.1%) of the participants were found to be O. The next highest group was A with frequency of 119(28.5%). Concerning ABO and Rh D blood group distribution from total subjects with O, A, B, and AB blood group 2.1%, 1.9%, 1.2%, and 1% were found Rhesus D-negative blood phenotype, respectively (Fig. 1).

In this study, the overall prevalence of Rh D negative phenotype was found 6.2% among reproductive age women in Arba Minch Zuria district (Table 2).

**Table 2:** Rh blood group profile among reproductive age women in Arba Minch Zuria district (n = 417)

Rhesus D blood group	Frequency (n)	Percentage (%)
Positive	391	93.8
Negative	26	6.2

## Discussion

The main purpose of this study was to assess the Rh D negative phenotype among reproductive age women. Rhesus incompatibility has got low attention or is often neglected in different parts of the world including developing countries like Ethiopia. Albeit it is cheap and easy to detect Rh D factor during pregnancy, the reproductive risk of Rh D negative women in Africa, Asia, or China is three times that of European women [15].

The distribution of Rh D negative varies widely across the world; among Caucasians, its prevalence is greater than 14% [16], whereas among different ethnic groups of sub-Saharan Africa its prevalence ranges between 2.4 and 4.5% [17–20].

This study showed that 159 (38.1%) of the total study participants were found to be blood group O. The next highest group was A with 119 (28.5%). This study revealed that 2.1%, 1.9%, 1.2%, and 1% of the study population with blood groups O, A, B, and AB were Rh D negative, respectively. It was noticed that 26 (6.2%) of the total study population was found Rh D negative.

This finding is in line with the study conducted in Jimma, Southwestern Ethiopia, which reported 6.3% Rh negativity among mothers [21]. The current finding is lower than the report of a study done in Gambella, Southwest Ethiopia, which reported that 19.37% Rh D negativity [22]. A study conducted at Mekelle; Northern Ethiopia showed that 8.8 of women were Rh D negative [23]. A research finding from Jimma, Ethiopia, reported a 7.2% Rh D negative phenotype among women [24]. This indicates Rh D-negative distribution among women varies in different parts of Ethiopia. Evidence showed that apart from the spatial and ethnic or racial variations, the Rh D-negative frequency varies temporally in a single population in different regions of the same country [25].

Studies conducted in southwestern and south-eastern Nigeria revealed a 5.5 and 4.5% Rh D negative factor among reproductive women [26, 27]. The current study revealed a high prevalence of Rh D-negative blood phenotype among women in the Arba Minch Zuria district. This study has some limitations which have to be taken into consideration while interpreting the findings. Since the study design was cross sectional, it does not confirm the cause-and-effect relationship. The current study has not checked weak D antigens because of resource limitation. However, to the best of our knowledge, this is the first study assessed Rh D factor exclusively among reproductive-age women at the community level in Ethiopia and therefore it can be used as a baseline for further researches.

## Conclusions and recommendations

This study showed a high prevalence of Rh D negative factor among women in Arba Minch Zuria district. Therefore, counseling of reproductive age women on the importance of Rh D factor status determination would be worthy to avoid the potential risk of sensitization among Rh D negative women in order to prevent hemolytic disease of the fetus and newborn.

## Data Availability

The data used to support the findings of this study are available from the corresponding author upon request.

## Abbreviations

CSA: central statistics agency
Rh: Rhesus
SPSS: Statistical Package for Social Science
FMH: feto-maternal haemorrhage
Rh D: Rhesus D factor
